# A Crude Extract Preparation and Optimization from a Genomically Engineered *Escherichia coli* for the Cell-Free Protein Synthesis System: Practical Laboratory Guideline

**DOI:** 10.3390/mps2030068

**Published:** 2019-08-09

**Authors:** Jeehye Kim, Caroline E. Copeland, Sahana R. Padumane, Yong-Chan Kwon

**Affiliations:** 1 Department of Biological and Agricultural Engineering, Louisiana State University, Baton Rouge, LA 70803, USA; 2 Louisiana State University Agricultural Center, Baton Rouge, LA 70803, USA

**Keywords:** cell-free protein synthesis, *E. coli* crude extract preparation, genomically engineered *E. coli*, sonication

## Abstract

With the advancement of synthetic biology, the cell-free protein synthesis (CFPS) system has been receiving the spotlight as a versatile toolkit for engineering natural and unnatural biological systems. The CFPS system reassembles the materials necessary for transcription and translation and recreates the in vitro protein synthesis environment by escaping a physical living boundary. The cell extract plays an essential role in this in vitro format. Here, we propose a practical protocol and method for *Escherichia coli*-derived cell extract preparation and optimization, which can be easily applied to both commercially available and genomically engineered *E. coli* strains. The protocol includes: (1) The preparation step for cell growth and harvest, (2) the thorough step-by-step procedures for *E. coli* cell extract preparation including the cell wash and lysis, centrifugation, runoff reaction, and dialysis, (3) the preparation for the CFPS reaction components and, (4) the quantification of cell extract and cell-free synthesized protein. We anticipate that the protocol in this research will provide a simple preparation and optimization procedure of a highly active *E. coli* cell extract.

## 1. Introduction

The technology involving the disruption of bacterial cells and collection of ribosomes for synthesizing proteins was first introduced when the fraction of ribosomes was identified as the core of the protein synthesis machinery of the cells [1]. The cell-free protein synthesis (CFPS) system has been developed for exclusive protein synthesis utilizing active ribosomes and other cellular machinery outside of the living cell [2,3]. Recent progress of synthetic biology highlights this versatile system as an essential toolkit for exploring and maneuvering complex cellular processes to accelerate technology advances [4,5]. The CFPS system offers many advantages over a cell-based system, such as ease of manipulating biochemical pathways [6,7], higher tolerance on chemicals and toxic compounds [8,9], utilization of PCR amplified linear template allowing for high-throughput preparation and protein synthesis of the gene of interest and breadboarding synthetic biological circuit [10,11,12,13], and the capability of highly efficient non-standard amino acid incorporation [14,15]. In addition, the CFPS system allows the benefits of unprecedented logistics along with freeze-drying paper-based format [16].

Since the cell extract carries most of the cellular machinery, its preparation is considered as the first important step for building a highly productive CFPS system. Many studies streamlined the overall procedure for cell extract preparation to improve overall extract performance in CFPS system [17,18,19,20,21]. Although recent progress in the preparation of *Escherichia coli* cell extract has resulted in an increase in protein productivity up to 1–1.5 mg/mL in a single batch cell-free system [22], the total protein yields are varied from strain to strain due to the necessary variations, dictated by the strain, of the three major cell extract preparation stages: pre-lysis, lysis, and post-lysis. The preparation step of the cell extract is crucial, as it contains key components for synthesizing proteins, so it is important to practice the optimized cell-free extract method for each *E. coli* strain for high protein yield. For example, Kim et al. [19] demonstrated that *E. coli* strain BL21(DE3)-derived cell extract prepared in a simpler procedure were more effective than the cell extracts prepared by the conventional method described by Pratt et al. [23]. However, the modified procedure was not as productive for the traditional host organism, *E. coli* A19 derived extract [19]. This evidence indicated that different preparation conditions are required depending on the *E. coli* strain of choice to maximize cell extract performance. However, the recent study from Kwon and Jewett was the first to introduce an optimized systemic cell extract preparation process for the non-commercial engineered *E. coli* strain K12 MG1655 (C495) which greatly advanced the potential for use in future biomedical/industrial applications [22]. In addition, the systemically optimized CFPS is inspiring novel ways to utilize the cell extracts from engineered *E. coli* strains for applications involving unnatural amino acid incorporation [15], patient-specific therapeutic vaccines [24], anticancer protein production [25], and more.

Here we describe a procedure for cell extract preparation step-by-step for the genomically engineered *E. coli* strain *ΔprfA ΔendA Δrne* [15] to generate the optimal cell extract with the maximum protein productivity. This protocol also can be applied to other strains with slight modification. The protocol aims to clarify which processing variables are the most critical for the cell extract performance during CFPS and how the processing condition can be optimized for different *E. coli* strains.

## 2. Experimental Design

**Figure 1:**
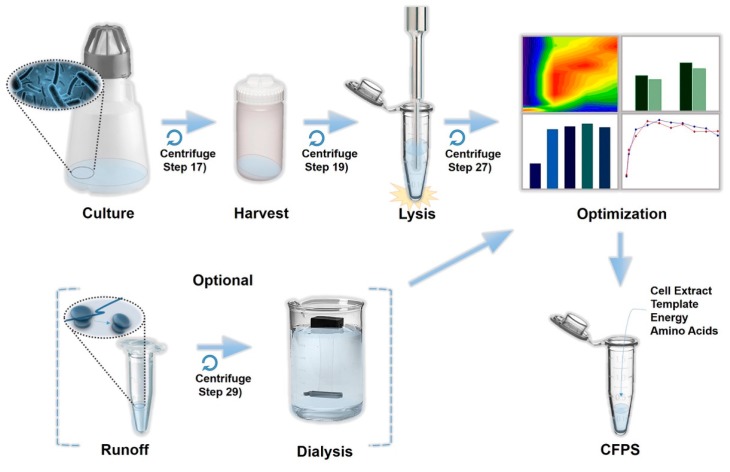
A workflow of the cell extract preparation and optimization.

The protocol in this study was designed to obtain the optimal cell extract that can maximize protein production yield during the cell-free protein synthesis. As illustrated in Figure 1, the streamlined processing steps were considered to be the most important parameters that influence the overall activity of cell extract: Pre-lysis (culture and harvest), lysis, and post-lysis (run-off reaction and dialysis) steps. Briefly, the procedures consist of three parts: (1) Tuning the cell culture and harvest time to obtain the most actively growing cells, (2) characterizing cell lysis condition by evaluating the relative protein production yield of each cell extract across sonication energy input and processing volume, and (3) an optional step of conducting a run-off reaction and dialysis for clearing out the cell extract [22,26]. This protocol will provide a practical guideline to produce a highly active cell extract by describing the overall extract preparation processes in detail for a better understanding of the CFPS system.

### 2.1. Materials

#### 2.1.1. *E. coli* Cell Preparation: Culture Media (2xYTPG and LB) and LB Agar Plate

- Genomically engineered *E. coli* strain K12 MG1655 ΔprfA ΔendA Δrne [15]
- Ampicillin sodium salt (Fisher Bioreagents, Fair Lawn, NJ, USA)
- Bacto^TM^ Tryptone (BD Biosciences, San Jose, CA, USA)
- Bacto^TM^ yeast extract (BD Biosciences)
- Bacto^TM^ agar (BD Biosciences)
- Glucose (Fisher Bioreagents)
- Sodium chloride (Fisher Bioreagents)
- Potassium phosphate dibasic (K_2_HPO_4_) (Fisher Bioreagents)
- Potassium phosphate monobasic (KH_2_PO_4_) (Fisher Bioreagents)
- Potassium hydroxide (Fisher Bioreagents)

#### 2.1.2. Cell Disruption and Extract Preparation: Buffer A and Dialysis

- 1,4-Dithio-DL-threitol (DTT) (Sigma-Aldrich, St. Louis, MO, USA)
- Potassium acetate (Fisher Bioreagents)
- Magnesium acetate tetrahydrate (Fisher Bioreagents)
- Tris base (Fisher Bioreagents)
- Potassium hydroxide (Fisher Bioreagents)
- Slide-A-Lyzer™ G2 dialysis cassette, 10K MWCO, 3 mL (Thermo Fisher Scientific, Waltham, MA, USA) for dialysis (if necessary)

#### 2.1.3. Cell-Free Protein Synthesis

- Magnesium glutamate (Sigma-Aldrich)
- Ammonium glutamate (MP Biomedicals, Santa Ana, CA, USA)
- Potassium glutamate (Sigma-Aldrich)
- Adenosine triphosphate (ATP) (Alfa Aesar, Haverhill, MA, USA)
- Cytidine triphosphate (CTP) (Alfa Aesar)
- Uridine triphosphate (UTP) (Alfa Aesar)
- Guanosine triphosphate (GTP) (Sigma-Aldrich)
- L-5-formyl-5, 6, 7, 8-tetrahydrofolic acid (Folinic acid) (Alfa Aesar)
- *E. coli* total tRNA mixture (from strain MRE600) (Roche Applied Science, Indianapolis, IN, USA)
- Phosphoenolpyruvate (PEP) (Alfa Aesar)
- 20 amino acids (Alfa Aesar)
- Nicotinamide adenine dinucleotide (NAD) (Thermo Fisher Scientific)
- Coenzyme-A (CoA) (Sigma-Aldrich)
- Potassium oxalate (Oxalic acid) (Alfa Aesar)
- Putrescine (Thermo Fisher Scientific)
- Spermidine (Thermo Fisher Scientific)
- HEPES (Thermo Fisher Scientific)
- Phusion^®^ high-fidelity DNA polymerase (New England Biolabs, Ipswich, MA, USA) for the preparation of PCR amplified linear template

#### 2.1.4. Protein Quantification

- NuPAGE^®^ 4–12% bis-tris gel (Thermo Fisher Scientific)
- NuPAGE^®^ LDS sample buffer (Thermo Fisher Scientific)
- NuPAGE^®^ MES SDS running buffer (20×) (Thermo Fisher Scientific)
- SimplyBlue^TM^ SafeStain (Thermo Fisher Scientific)
- Coomassie Blue assay reagent (Thermo Fisher Scientific)
- 1,4-Dithio-DL-threitol (DTT) (Sigma-Aldrich)

### 2.2. Equipment

- 2.5 L baffled Tunair shake flasks (IBI Scientific, Peosta, IA, USA)
- 300 mL baffled Tunair shake flasks (IBI Scientific)
- New Brunswick™ Innova^®^ 42 incubator shaker (Eppendorf, Hamburg, Germany)
- Genesys^TM^ 6 UV-Vis spectrophotometer (Thermo Fisher Scientific)
- Sorvall Legend X1 Sorvall Legend X1R centrifuge (Thermo Fisher Scientific, USA)
- TX-400 swinging bucket rotor (Thermo Fisher Scientific)
- Round Buckets for TX-400 rotor (Thermo Fisher Scientific)
- Fiberlite™ F15-8 × 50cy fixed angle rotor (Thermo Fisher Scientific)
- Thermo Scientific set of four adapters for 15 mL Conical Tube (Thermo Fisher Scientific)
- MicroClick 30 × 2 fixed angle microtube rotor (Thermo Fisher Scientific)
- Q125 Sonicator with 1/8” (3 mm) diameter probe (Qsonica, Newtown, CT, USA)
- Synergy^TM^ HTX multi-mode microplate reader (BioTek, Winooski, VT, USA)
- Genesys^TM^ 6 UV-Vis spectrophotometer (Thermo Fisher Scientific)
- Fisherbrand™ accuSpin™ micro 17 microcentrifuges (Thermo Fisher Scientific)
- PowerPac™ basic power supply (Bio-Rad, Hercules, CA, USA)
- Mini gel tank (Invitrogen, Carlsbad, CA, USA)
- Direct-Q3^®^ UV water purification system (Millipore, Burlington, MA, USA)
- Scotsman flake ice maker (CurranTaylor, Canonsburg, PA, USA)

## 3. Procedure

### 3.1. Cell Extract Preparation. Time for Completion: Four Days

#### 3.1.1. Day 1

(1) LB media: Dissolve 10 g/L of tryptone, 5 g/L of yeast extract, 10 g/L of sodium chloride in Milli-Q water and sterilize at 121 °C for 30 min.
(2) LB-agar plate: Dissolve 10 g/L of tryptone, 5 g/L of yeast extract, 10 g/L of sodium chloride, and 15 g/L of bacto-agar in Milli-Q water and sterilize 121 °C for 30 min. Place the container in 55–65 °C water bath to cool the solution. Add appropriate antibiotics (if necessary), mix well, and solidify in Petri dishes (20 mL each).
(3) Streak LB-agar plate with *E. coli* K12 MG1655 *ΔprfA ΔendA Δrne* and incubate overnight (16–20 h) at 34 °C.
(4) Put 1 L of Milli-Q water and centrifuge rotors in 4 °C.
(5) Prepare three buffer A stock solutions (100x) in separate bottles (500 mL each), (a) 1 M Tris-acetate (pH 8.2): Dissolve 60.57 g of Tris base in 500 mL of Milli-Q water and adjust pH to 8.2 with 5 N potassium hydroxide, (b) 1.4 M Magnesium acetate: dissolve 107.23 g of magnesium acetate tetrahydrate in 500 mL of Milli-Q water, (c) 6 M potassium acetate: Dissolve 294.42 g of potassium acetate in 500 mL of Milli-Q water. All solutions are filtrated by passing them through a 0.2 μm filter unit.

#### 3.1.2. Day 2

(1) Pick a single bacterial colony (2–3 mm in diameter) from the plate and transfer the colony into 30 mL of LB media in a 125 mL baffled flask with appropriate antibiotics.
(2) Incubate the culture for overnight (8–12 h) at 34 °C with vigorous shaking at 250 rpm.
(3) Glucose solution: 18 g/L of glucose (0.4 M) in Milli-Q water.
(4) 2xYTP media: dissolve 16 g of tryptone, 10 g of yeast extract, 5 g of sodium chloride, 7 g of potassium phosphate dibasic, and 3 g of potassium phosphate monobasic in 500 mL of Milli-Q water in 1 L beaker.
(5) Adjust pH with 5 N potassium hydroxide until it reaches 7.2 and then add Milli-Q water until it reaches 750 mL.
(6) Transfer 750 mL 2xYTP (pH 7.2) to 2.5 L baffled Tunair shake flask, close the cap, and warp with aluminum foil.
(7)
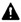
**CRITICAL STEP** 2xYTP media and glucose solution are prepared in separate containers and sterilize at 121 °C for 30 min separately.

#### 3.1.3. Day 3

(8) Add 250 mL of sterilized 0.4 M glucose solution into 750 mL of sterilized 2xYTP media near the flame or in the biosafety cabinet right before inoculation. Shake well. The total 2xYTPG media volume is 1 L. Antibiotics are not included.
(9) Transfer 10 mL of overnight culture (1:100 ratio) to 1 L main culture media (step (11)) near the flame or in the biosafety cabinet.
(10)
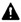
**CRITICAL STEP** Monitor the cell growth rate (OD_600_) including initial inoculum by spectrophotometer until OD_600_ reaches to the mid-exponential growth phase. Calculate cell doubling time (*T_d_*). Optimal doubling time is 35.6 ± 0.3 [15]
(11)
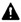
**CRITICAL STEP** From here, the cells need to handle at 4 °C or below. Put the centrifuge bottles in ice before harvest. Weigh empty 50 mL conical tubes and mark weight (g) on the tube and put in ice. Turn on the centrifuge and set the temperature at 4 °C.
(12) Harvest the cell when the growth curve reaches at a mid-exponential phase by centrifugation (TX-400 swinging bucket rotor) at 5000 RCF at 4 °C for 15 min and then discard the supernatants.
(13) Prepare buffer A solution: Combine 10 mL of each stock solutions with 970 mL of 4 °C chilled Milli-Q water (from Day 1) and add 1 mL of 1 M DTT (Table 1). Mix well and put in ice.
(14) Cell washing: transfer the harvested cells to 50 mL conical tube and add chilled buffer A solution up to 40 mL. Resuspend the cells by shaking or vortexing. 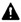 **CRITICAL STEP** Maintain low temperature during washes. Centrifugation (Fiberlite™ F15-8 × 50cy fixed angle rotor) at 5000 RCF at 4 °C for 10 min and then discard the supernatants. Repeat three times.
(15)
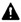
**CRITICAL STEP** Weigh wet cell weight (g) and marked on the conical tube.
(16)
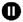
**PAUSE STEP** Flash freeze the pelleted cells in liquid nitrogen and stored at −80 °C until the next step.

#### 3.1.4. Day 4 (or Continue Day 3)

(17) Take out frozen cells from −80 °C and thaw in ice.
(18) Resuspend the cells in 1 mL of buffer A per 1 g of wet cell mass.
(19)
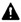
**CRITICAL STEP** Transfer the cell suspension into 1.5 mL microtube in an ice-water container to minimize heat damage during sonication.
(20) The cell suspension is lysed using sonicator at a frequency of 20 kHz and 50% amplitude. Different levels of energy input (Joules) and sample volumes are applied during sonication to obtain the highest protein production yield for CFPS reaction.
(21)
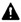
**CRITICAL STEP** Add DTT into cell lysate (3 µL per 1 mL lysate), invert several times quickly, and place the microtube in the ice-water bucket until the next step.
(22) The lysate is centrifuged once at 12,000 RCF at 4 °C for 10 min.
(23)
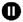
**PAUSE STEP** Transfer the supernatant (crude cell extract) to a fresh microtube, flash freeze in liquid nitrogen, and store at −80 °C until use.
(24) **OPTIONAL STEP** The runoff reaction (pre-incubation at 37 °C at 250 rpm) is performed in different reaction time (0, 20, 40, 60, and 80 min). After runoff reaction, clear the cell extract by centrifugation at 10,000 RCF at 4 °C for 10 min.
(25) **OPTIONAL STEP** The cell extract can be dialyzed if necessary. Cell extract transfer to dialysis cassette (Slide-A-Lyzer™ G2 dialysis cassette, 10K MWCO) and dialyze in chilled buffer A with stirring at 4 °C. Buffer A is exchanged every 45 min four times.

### 3.2. Cell-Free Protein Synthesis. Time for Completion: One to Two Days

(1) The standard CFPS reaction mixture (15 µL) consists of following components: 12 mM magnesium glutamate, 10 mM ammonium glutamate, 130 mM potassium glutamate, 1.2 mM ATP, 0.85 mM each of GTP, UTP, and CTP, 34.0 µg/mL folinic acid, 171 µg/mL *E. coli* total tRNA (strain MRE600), 2 mM each of 20 amino acids, 33 mM phosphoenolpyruvate (PEP), 0.33 mM nicotinamide adenine dinucleotide (NAD), 0.27 mM coenzyme-A (CoA), 1.5 mM spermidine, 1 mM putrescine, 4 mM sodium oxalate, 57 mm HEPES-KOH (pH 7.5), 100 µg/mL T7 RNA polymerase, 13.3 ug/mL pJL1-sfGFP plasmid and 27% v/v of cell extract. All components store at −80 °C until use.
(2) Place dry heat block (filled with water) in the incubator. Set temperature at 30 °C or 37 °C.
(3)
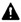
**CRITICAL STEP** Take out all CFPS components from the freezer and thaw in ice. Prepare fresh microtubes for the CFPS reaction in ice.
(4) Vortex all CFPS reaction components well except for T7 RNA polymerase, cell extract, and plasmid. To mix T7 RNA polymerase, cell extract, and plasmid gently flick the tube.
(5)
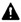
**CRITICAL STEP** Mix all reaction components in a fresh microtube and pipetting up and down to mix homogeneously and minimize bubbles.
(6) Briefly spin down the microtubes.
(7) Put the microtubes in the dry heat block and incubate for the desired time period (20 h for plasmid, 4 h for PCR amplified linear template).
(8) Put all CFPS reaction components back in the freezer for next use.

### 3.3. Protein Quantification

#### 3.3.1. Superfolder Green Fluorescent Protein (sfGFP)—Fluorescence. Time for Completion: 30 min

(1) After the CFPS reaction, briefly vortex microtube.
(2) Aliquot 48 µL of Milli-Q water in 96-well flat bottom black half-well microplate and add 2 µL of cell-free synthesized protein into each well to bring the total volume to 50 µL.
(3) Place the microplate in the plate reader and set it to 15 s orbital shaking (fast) and measure the fluorescence. Excitation and emission wavelengths are 485 and 528 nm, respectively.

#### 3.3.2. SDS-PAGE. Time for Completion: 3 h

(1) Set the dry heat block at 80 °C.
(2) Mix 5 µL of the cell-free synthesized sample, 5 µL of 4x sample loading dye, and 10 µL of 200 mM DTT solution to bring the total volume to 20 µL.
(3) Vortex well and incubate 5 min in 80 °C dry heat block.
(4) Prepare 4–12% SDS-PAGE, SDS-PAGE loading buffer, and gel tank. Clear out the gel wells by injection of the loading buffer.
(5) After 5 min of protein denaturation, spin down and vortex the samples.
(6) Load 10 µL of samples mixture to each well. For the size determination, 5 µL of pre-stained protein ladder is loaded to the first well.
(7) Run the gel electrophoresis with the voltage 150 V for one hour.
(8) Cell-free synthesized proteins are visualized by Coomassie blue staining.

#### 3.3.3. Total Protein—Bradford Assay. Time for Completion: 30 min

(1) Prepare the BSA dilutes (2 mg/mL to 25 µg/mL) for standards and use ultra-pure water as a blank (0 µg/mL).
(2) The original cell extracts are pre-diluted 40-fold in ultra-pure water (i.e., 5 µL of cell extract plus 195 µL of water) and then use as test samples for the protein assay.
(3) Mix the protein-dye solution with standards or samples, incubate for 10 min at room temperature, and measure the absorbance at 595 nm.
(4) The original (undiluted) concentration is determined by multiplying 40 to the concentration of the diluted test sample.
(5) The amount of total protein in the cell extracts are ranged from 15.4 to 54.8 mg/mL depends on the sonication energy input and volume.

## 4. Expected Results

For many years, the procedure described by Pratt [23] has been considered as the standard method for preparation of *E. coli* cell extract. However, this standard process requires a lengthy preparation time in combination with multiple steps, which can cause complications when scaling up the cell-extract resulting in the inconsistent protein productivity [27]. Over the decades, studies on cell extract preparation have progressed and accomplished to show strong evidence that the systemic optimization of key parameters can significantly increase the protein production yields through the cell-free protein synthesis reaction.

### 4.1. Pre-Lysis Procedure

Manipulating bacterial cell culture condition influences the cell’s physiological state and cellular composition over the course of its growth. Several chemical and physical parameters are considered as essential factors which can enhance metabolic properties during the culture, including culture media, temperature, pH, and oxygen. It is important to pay attention to the method that the cells are grown and harvested because these conditions directly impact the protein synthesis performance of the resulting cell extract during the CFPS reaction. For example, using the cell extract that was cultured in enriched media with inorganic phosphate and glucose in high concentration (2xYTPG) has shown improved protein production yield [28]. In addition, enhanced central catabolic pathway and tricarboxylic acid (TCA) cycle support energy regeneration during CFPS reaction [29,30]. Rapidly growing cells maintain an increased catabolic and anabolic protein synthesis balance with sufficient nutrition supplement, and intracellular mass, including ribosome and volume of a bacterial cell increase exponentially as well [31,32]. Collection of bacterial cells at the exponential growth is known to be critical for obtaining more active cell extracts. For example, Kwon and Jewett examined the increased activity of cell extract when the cells were harvested at the exponential phase by monitoring cell growth rate [22].

**Figure 2:**
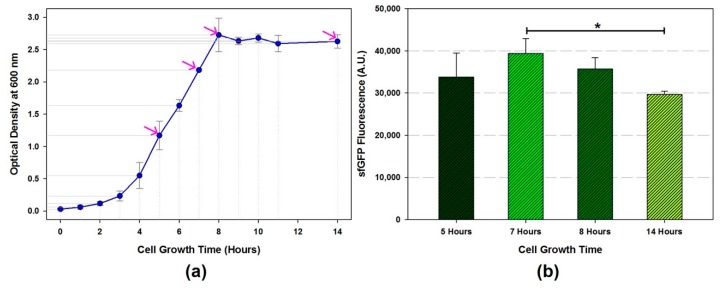
Cell extract performance upon the different cell harvest time. (**a**) The *E. coli* K12 MG1655 *ΔprfA ΔendA Δrne* growth curve. The cell collected at 5, 7, 8, 14 h from the beginning of cell culture. The pink arrows indicate each harvest time. (**b**) The intensity of the cell-free synthesized sfGFP fluorescence from each cell extract (1750 Joules of sonication energy input, 1000 µL of processing volume, and 60 min of runoff reaction time). Data are presented as the average ± standard deviation (*n* = 3); * *p* < 0.05 according to one-way analysis of variance (ANOVA).

In this study, one liter of 2xYTPG culture media was used for *E. coli* K12 MG1655 *ΔprfA ΔendA Δrne* cell culture. Overnight cultured cells (20 mL in LB) were inoculated to the 2xYTPG main culture media. Initial OD_600_ was measured from the overnight cultured cell. The cells were collected over the course of the culture, early mid-exponential phase, mid-exponential phase, early stationary phase, and stationary phase, which are marked with the pink arrows in Figure 2a. The cell extract was prepared with optimized conditions (sonication energy and cell-buffer volume) described previously [22]. The CFPS reaction with each cell extract was examined to determine the best harvest time point for maximizing protein productivity. Notably, the cell extract harvested at the mid-exponential growth phase (7 h culture time) showed significantly increased protein productivity (32.8%) compared to that of the extract harvested in the stationary growth phase (Figure 2b). This result indicated that the performance of cell-extract imperatively depends on the contents of intracellular macromolecules, which change during cell growth. Determination of mid-exponential growth for the cell collection is the first step to obtain the highly active cell extract for the CFPS reaction.

### 4.2. Lysis

Kwon and Jewett developed a method to determine the proper level of sonication energy input per sample volume, which is required to disrupt *E. coli* cell wall without damaging the intercellular components. This cell extract showed the overall same protein production capacity compared with already established cell extract preparation methods [22].

**Figure 3:**
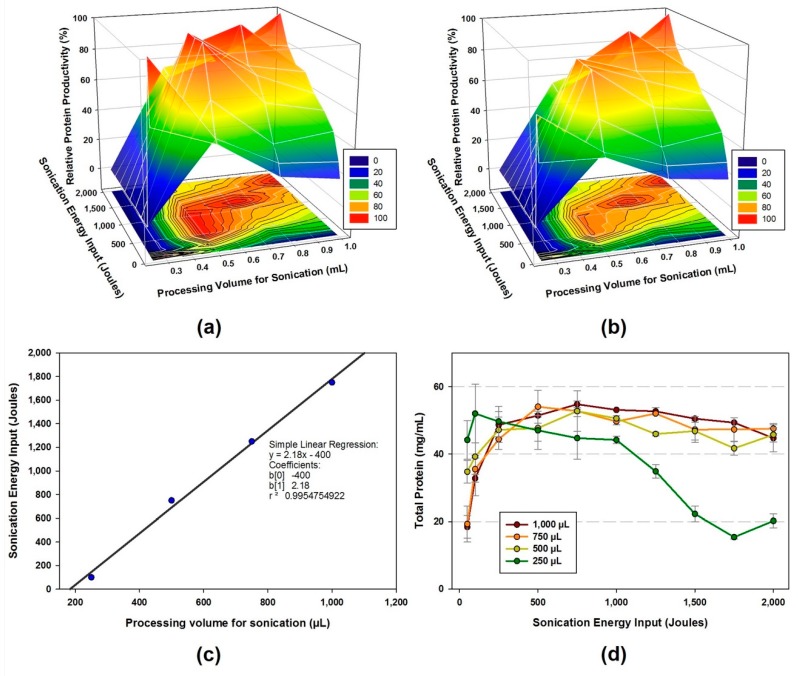
The effect of total energy input and cell suspension volume on the protein productivity of cell extract. (**a**) The relative protein productivity per each volume. The productivity was represented from 0 to 100% at each processing volume (250, 500, 750, and 1000 µL) by different energy input (from 50 J to 2000 J) to find the optimal sonication energy input for each processing volume. (**b**) The relative protein productivity with all processing volume (250 to 1000 µL, combined) was represented from 0 to 100% by different energy input (from 50 J to 2000 J) to find the optimal sonication energy and volume for the highest protein productivity. (**c**) A simple linear regression model of processing volume and sonication energy input. The sonication energy input resulting in the highly active cell extract was selected for each processing volume (250, 500, 750, and 1000 µL) and plotted in a regression model. From the regression model, the optimal energy for each processing volume can be calculated as follows: (Sonication energy input (Joules)) = (The designed processing volume for sonication (µL) + 400) 2.18^−1^. (**d**) The changes in the total protein amount of the cell extract in different sonication energy input. Data are presented as the average ± standard deviation (*n* = 2).

In this study, the optimal lysis condition was determined with two independent variables: the processing volume for sonication and sonication energy input as described previously [22]. The aim of this study is to investigate the variations in the protein productivity of cell extracts, which is the result of the combinatorial change of the processing volume and sonication energy input. First, ten different sonication energy levels (50, 100, 250, 500, 750, 1000, 1250, 1500, 1750, and 2000 Joules) were applied to each of four different processing volumes (250, 500, 750, and 1000 µL) to determine the optimal cell lysis condition during sonication. The relative productivity of the cell extract was determined by the fluorescence intensity of the cell-free synthesized protein, sfGFP, divided by the fluorescence intensity from the cell extract showing the highest intensity at each processing volume. Then the relative intensity was represented as 0 to 100% for processing volume. The 3D mesh plot indicates the maximum protein productivity—color represents relative productivity—at each volume of cell extract tested against the sonication energy input resulting in the optimal conditions listed here: 100 J of sonication energy input for 250 µL, 500 to 1000 J for 500 µL, 1250 J for 750 µL, and 1750 J for 1000 µL (Figure 3a). Notably, 500 µL of processing volume showed a wide range of sonication energy input. Since the observed optimal sonication energy input at each processing volume ranged from 250 to 1000 µL fits the linear regression model in Figure 3c, the optimal sonication energy input for any processing volume ranged between 250 and 1000 µL can be predicted to obtain the best performing cell extract. The relative protein productivity is listed in Supplementary Table S1. The optimal sonication input for processing volume can be projected using the linear regression model as follows: y=2.18x−400 where x is the designed processing volume for sonication. The cell-free synthesized sfGFP from the cell extract variants of processing volume 750 µL were analyzed by Coomassie Blue staining after running the reaction samples on a 4–12% Bis-Tris acrylamide gel (Supplementary Figure S1). Next, all the variations in the protein productivity of cell extracts from the combined alterations of sonication energy input and processing volume were profiled. The cell extract lysed with the 1750 Joules of energy input and the 1000 µL of processing volume showed the overall highest protein productivity among the forty energy–volume combinations (Figure 3b). The relative productivity of cell extract was determined by the fluorescence intensity of the cell-free synthesized sfGFP divided by the sfGFP fluorescence intensity from the cell extract showing the highest intensity in all combined modifications. The relative intensity (combined) was represented as 0 to 100%. The result indicated that the higher volumes, such as 750 and 1000 µL of the processing volume tend to be more favorable to obtain more active cell extract when combined with the optimal sonication energy input (Supplementary Table S2). The linear relationship between sonication volume and energy input was plotted in Supplementary Figure S2. Lastly, we measured the total amount of protein in the cell extract. The total protein concentration in the cell extract increased by up to 54.78 mg/mL with the increase of sonication energy, and then showed a plateau in the range of 500–2000 Joules. This trend is clearly shown in the 500, 750, and 1000 µL of the processing volume (Figure 3d). Interestingly, the variation in the total protein of the cell extract was not parallel to the protein productivity of the cell extract (Supplementary Figure S3a–c). Although it is difficult to determine that the exact amount of required total protein in cell extract for the optimal protein production performance, approximately 45 to 50 mg/mL of total protein contents showed 60–100% of relative protein productivity (Supplementary Figure S3). The cell extract with low processing volume (250 µL) showed declining trends along with the increase of the sonication energy input (Figure 3d and Supplementary Figure S3d). Protein degradation by overheating is an inevitable drawback of the sonication process. To minimize possible heat degradation, we carried out sonication by 10 s lysis and 10 s cooling during the total input energy reached to our preset values in ice water as described previously [22]. Moreover, we assumed that the higher energy which is out of range of the optimal value is a lethal effect to the cell extract activity by disruption of all the macromolecule contents in the cell extract.

### 4.3. Post-Lysis Procedure

Runoff reaction and dialysis were considered as cell extract clarification steps in traditional cell extract preparation. However, commercially available *E. coli* strain BL21 showed robust cell extract performance without these additional processes after lysis [19]. Kwon and Jewett further optimized a correlation between runoff reaction time and cell extract performance for *E. coli* strains BL21(DE3) star and a partially engineered C495 [22]. Here, we investigated the effect of runoff reaction and dialysis on the protein productivity of cell extract derived from genomically engineered *E. coli* strain.

**Figure 4:**
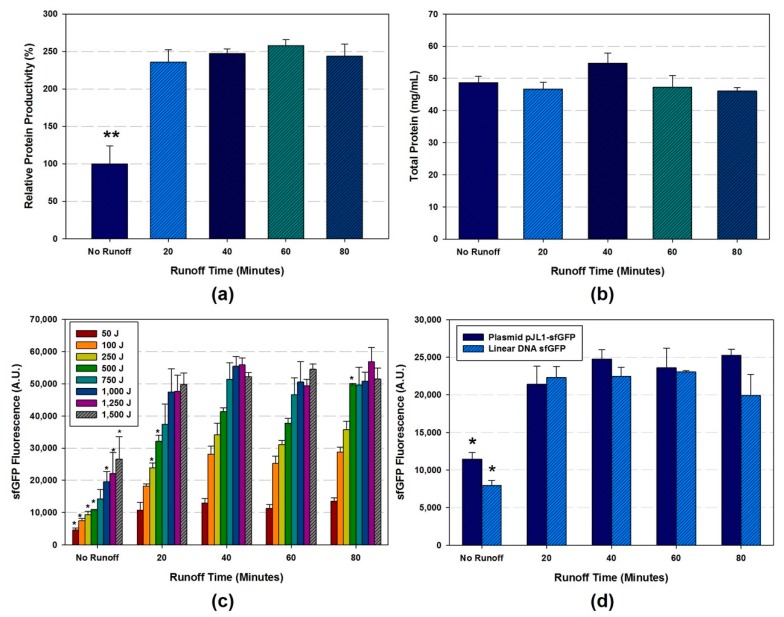
The effect of runoff reaction on protein productivity and total protein of the cell extract. (**a**) The relative protein productivity using the cell extract produced by different runoff reaction time ranged 0–80 min. Data depicted as the mean ± SD (*n* = 3); ** *p* < 0.001 according to one-way analysis of variance (ANOVA) (**b**) The total protein concentration in the cell extract treated with different runoff reaction time. For Figure 4a,b, the same cell lysate was used with the sonication condition of 1500 Joules of sonication input and 1000 µL of processing volume. (**c**) The effect of runoff at different lysis condition. All lysis procedure was fixed to 1000 µL of processing volume for sonication with different energy input ranged from 50 to 1500 Joules. Data depicted as the mean ± SD (*n* = 3), * *p* < 0.001 according to one-way analysis of variance (ANOVA). (**d**) CFPS reaction with runoff time variants on plasmid and PCR amplified linear template. Data depicted as the mean ± SD (*n* = 3), * *p* < 0.05 according to one-way analysis of variance (ANOVA).

The different runoff reaction was conducted after lysis and centrifugation. The CFPS reaction was carried out for 20 h at 30 °C with pJL1-sfGFP and PCR amplified linear sfGFP template. The data on protein productivity by different runoff time in Figure 4a,b shows that the runoff reaction time is essential for the cell extract from *E. coli* K12 MG1655 *ΔprfA ΔendA Δrne*. Protein production performance is comparable to the results in 40–80 min of runoff incubation time reported previously [21,22]. The statistical data using the one-way ANOVA indicates that there were significant differences (*p* < 0.001) between the cell extract with and without runoff reactions. However, there were no significant differences between cell extract within the 20–80 runoff reaction times. In addition, we observed that total protein contents were preserved during runoff reaction (Figure 4b). Next, we studied the effect of runoff reaction on all different sonication condition (50–1500 Joules, 1000 µL volume processing). The overall result is well correlated to our previous findings (Figure 4c and Supplementary Figure S3a–d). Regardless of sonication optimization, the significant increase of protein productivity was detected after runoff reaction *(p* < 0.001). Lastly, the effect of runoff reaction time using different DNA template was investigated. We carried out the CFPS reaction for 4 h for both PCR amplified linear template and plasmid instead of applying CFPS reaction for 20 h due to the instability of linear template. In both groups, the cell extract which was prepared without runoff reaction showed significantly low productivity (* p < 0.05) compared to the extract with runoff reaction (Figure 4d). Interestingly, there was no significant difference between the extracts with 20–80 min runoff reaction for the model genomically engineered *E. coli* strain.

**Figure 5:**
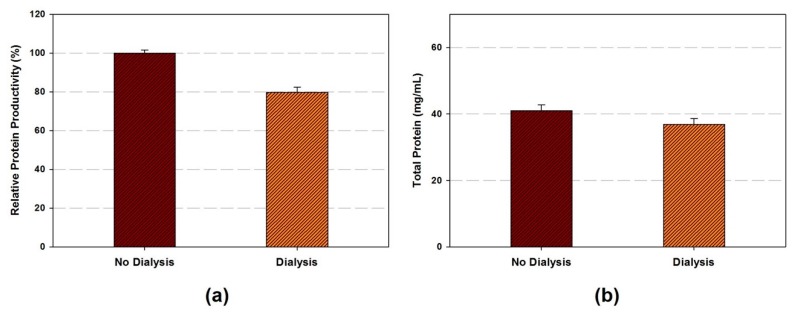
(**a**) The relative protein productivity, (**b**) total protein (mg/mL) of cell extract in the condition of dialysis. Data are presented as the average ± standard deviation (*n* = 2).

After the cell lysis, centrifugation, and runoff reaction, the remained byproduct can be cleared from the cell extract by dialysis. While the conventional cell extract preparation method included the dialysis step for final cell extract [18], more recently optimized cell extract preparation methods showed the same protein production performance without this step [19,22,33]. In this study, we applied the dialysis as the final optional step in the entire process. The cell extract prepared as followed: 1750 Joules of energy input, 1000 µL processing volume, and 60 min of runoff reaction. The dialysis was carried out with 2 mL of cell extract in a dialysis bag (10K MWCO) at 4 °C. Buffer A was replaced every 45 min for four times. Although total protein concentration is preserved after dialysis, protein productivity was decreased by 22% (Figure 5a,b).

## 5. Conclusions

Over the past decade, the cell-free system has been revitalized as an essential toolkit for synthetic biology and biotechnology research. Moreover, with its unique non-living feature, the cell-free system provides a new insight to understand the cellular processes outside the shell. Preparation of a highly active cell extract is the first step to build this versatile CFPS system. In this study, we discussed a detailed cell extract preparation protocol in three major stages encompassing cell culture, sonication, and optional post-lysis steps for genomically engineered *E. coli* K12 MG1655 *ΔprfA ΔendA Δrne* as a model strain. We expect that this protocol will provide not only a practical guideline but also a foundation for the entire CFPS system.

## Figures and Tables

**Table 1:** The components for 1 L of buffer A.

Reagents	Mixing Volume (mL)
1 M Tris-OAc, pH 8.2	10
1.4 M Mg(OAc)_2_	10
6 M KOAc	10
1 M DTT	1
Chilled Milli-Q water	970

## Supplementary Materials

The following are available online at https://www.mdpi.com/2409-9279/2/3/68/s1, Table S1: The relative protein productivity from the different sonication energy input at each processing volume, Table S2: The relative protein productivity with all processing volume (250–1000 µL, combined), Figure S1: The SDS-PAGE of the cell-free reaction mixture after reaction at 37 °C for 24 h, Figure S2: The linear relationship between sonication volume and energy input, Figure S3: The total protein (mg/mL) and relative sfGFP productivity (%) of cell extract variants of different sonication energy input ranged from 50 J to 2000 J.
