# Supplementary material for: A Crude Extract Preparation and Optimization from a Genomically Engineered Escherichia coli for the Cell-Free Protein Synthesis System: Practical Laboratory Guideline
Source: Methods Protoc. 2019 Aug 9;2(3):68. doi: 10.3390/mps2030068 (PMC6789667; doi:10.3390/mps2030068)
Supplement: Supplementary file 1 [file mps-02-00068-s001.pdf]

Supplementary materials

A Crude Extract Preparation and Optimization from a Genomically Engineered *Escherichia coli* for the Cell-Free Protein Synthesis System: Practical Laboratory Guideline

Jeehye Kim [1,†], Caroline E. Copeland [1,†], Sahana R. Padumane [1] and Yong-Chan Kwon [1,2,*]

[1] Department of Biological and Agricultural Engineering, Louisiana State University, Baton Rouge, LA 70803, USA

[2] Louisiana State University Agricultural Center, Baton Rouge, LA 70803, USA

* Correspondence: yckwon@lsu.edu; Tel: +1-225-578-4325

† These authors contributed equally to this work.

Contents:

Supplementary Table S1

Supplementary Table S2

Supplementary Figure S1

Supplementary Figure S2

Supplementary Figure S3

Supplementary Table S1. The relative protein productivity from the different sonication energy input at each processing volume. The productivity was represented from 0 to 100 % at each processing volume (250, 500, 750, and 1000 μL) by different energy input (from 50 J to 2,000 J) to find the optimal sonication energy input for each processing volume.

Sonication Energy (J)	Processing volume for sonication (μL)			
	**1000**	**750**	**500**	**250**
**2000**	86.51	67.69	66.25	0.32
**1750**	100.00	75.15	62.86	0.39
**1500**	81.04	85.25	74.61	0.40
**1250**	71.56	100.00	87.47	0.45
**1000**	70.91	77.38	100.00	0.47
**750**	60.90	74.98	99.46	0.61
**500**	54.36	62.03	100.00	0.39
**250**	45.66	41.73	70.83	0.44
**100**	29.86	32.95	51.20	100.00
**50**	20.09	25.62	48.78	61.96

Supplementary Table S2. The relative protein productivity with all processing volume (250 – 1000 μL, combined). The relative productivity was represented from 0 to 100 % by different energy input (from 50 J to 2,000 J) to find the optimal sonication energy and volume for the highest protein productivity.

Sonication Energy (J)	Processing volume for sonication (μL)			
	1000	750	500	250
**2000**	86.51	65.23	56.38	0.22
**1750**	100.00	72.42	53.50	0.27
**1500**	81.04	82.14	63.49	0.28
**1250**	71.56	96.36	74.44	0.31
**1000**	70.91	74.57	85.10	0.33
**750**	60.90	72.25	84.64	0.42
**500**	54.36	59.78	85.10	0.27
**250**	45.66	40.21	60.28	0.31
**100**	29.86	31.76	43.58	68.64
**50**	20.09	24.69	41.52	42.53

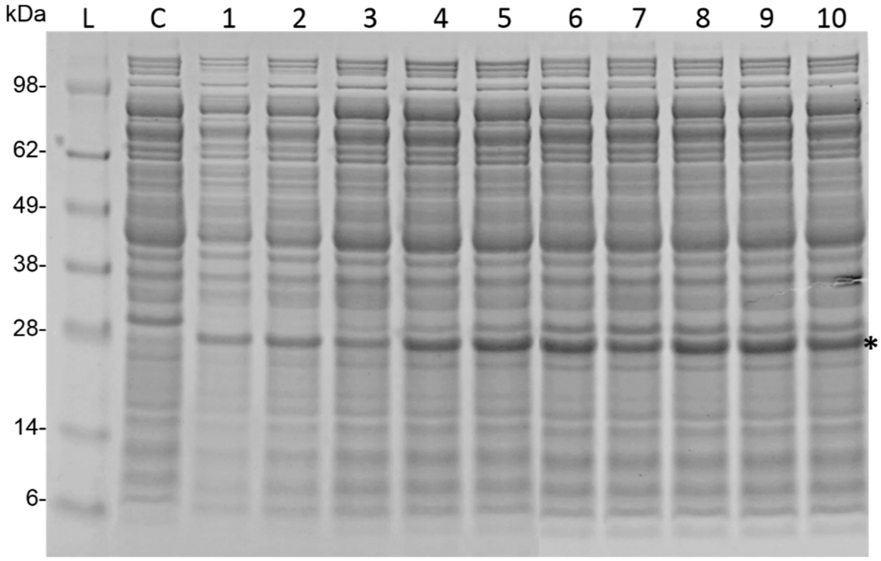

Supplementary Figure S1. The SDS-PAGE of the cell-free reaction mixture after reaction at 37 °C for 24 h. The amount of synthesized sfGFP protein from cell extract variants of different sonication energy input ranged 50 J to 2,000 J. Sonication processing volume was 750 μL. (L) protein ladder (C) Blank. cell-free reaction mixture without pJL1-sfGFP. (1) 50 J (2) 100 J (3) 250 J (4) 500 J (5) 750 J (6) 1000 J (7) 1250 J (8) 1500 J (9) 1750 J (10) 2000 J. Numbers next to the molecular weight ladder (L) represent the approximate kilodalton (kDa) size of the band.

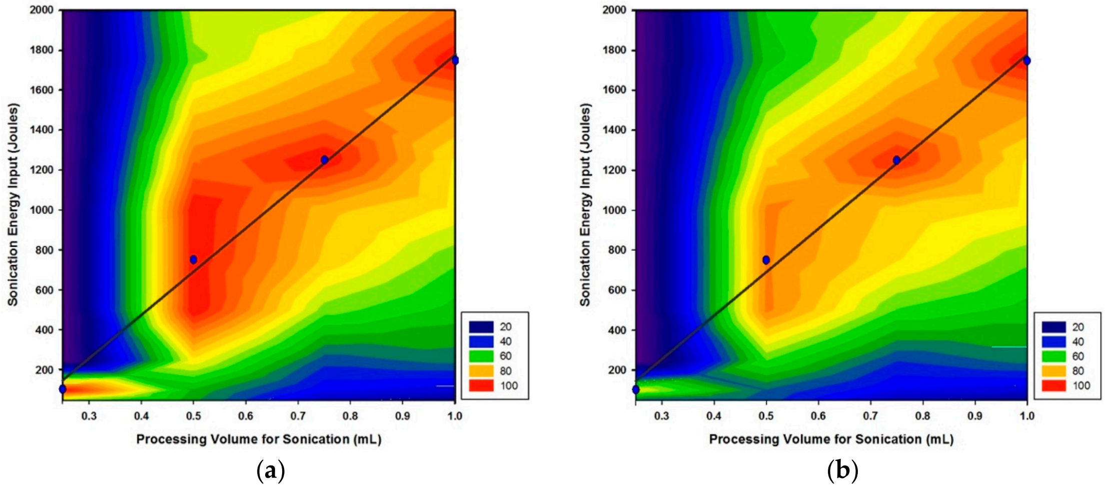

Supplementary Figure S2. The linear relationship between sonication volume and energy input. The relative protein productivity in different sonication input (50 J to 2000 J) and processing volume (250 µL to 1000 µL) The total protein (mg/mL) and relative sfGFP productivity (%) of cell extract variants of different sonication energy input ranged 50 J to 2,000 J. **(a)** The relative protein productivity (0 – 100 %) per each volume. **(b)** The relative protein productivity with all processing volume (250 to 1000 µL, combined) was represented from 0 to 100 %.

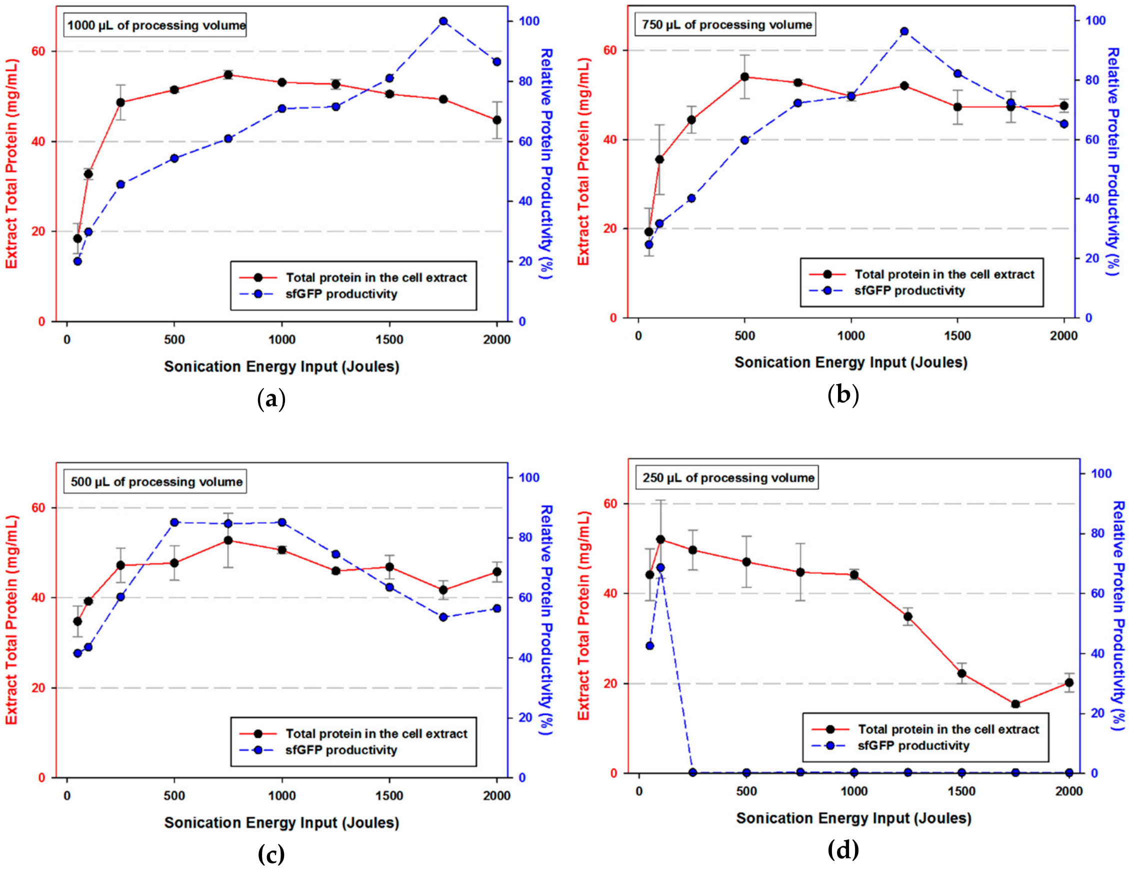

Supplementary Figure S3. The total protein (mg/mL) and relative sfGFP productivity (%) of cell extract variants of different sonication energy input ranged from 50 J to 2,000 J. **(a)** with the 1,000 µL of processing volume for sonication. **(b)** With the 750 µL of processing volume for sonication. **(c)** With the 500 µL of processing volume for sonication. **(d)** With the 250 µL of processing volume for sonication. Data for cell extract total protein are presented as the average ± standard deviation (n = 2).
